# Exploring physical and functional EEG connectivity with multilayer graph transformer convolutional networks for emotion recognition

**DOI:** 10.3389/fnhum.2025.1715410

**Published:** 2026-01-12

**Authors:** S. M. Atoar Rahman, Md Ibrahim Khalil, Hui Zhou, Ziyun Ding, Yu Guo

**Affiliations:** 1 School of Automation, Nanjing University of Science and Technology, Nanjing, Jiangsu, China; 2 School of Engineering, University of Birmingham, Birmingham, United Kingdom

**Keywords:** EEG emotion recognition, graph transformer convolutional layers, graph convolutional neural networks, physical proximity, functional connectivity

## Abstract

Electroencephalogram (EEG)-based emotion recognition has emerged as a compelling direction in affective computing, driven by its ability to provide objective, neural-level insights into emotional states. However, the high-dimensional and complex spatial and functional characteristics of EEG data present substantial challenges for accurate modeling. To address this, we propose Multilayer-GTCN (Multilayer Graph Transformer Convolutional Network), which combines the strengths of Graph Convolutional Networks (GCNs) and Graph Transformer layers to effectively capture both local and global dependencies in EEG signals. The framework employs a dual-graph design over feature nodes: a physical proximity graph instantiated as a complete topology to stabilize information flow, and a functional connectivity graph whose edges are correlations derived from inter-feature relationships. Within this representation, GCN layers consolidate stable relational patterns, while transformer-based graph convolutions capture long-range dependencies and transient interactions across the feature space. Combining the two encoded views results in representations that jointly capture localized structure and global context, providing a robust basis for affective decoding. Extensive experiments on benchmark datasets confirm the effectiveness of our approach, achieving 98.24 ± 1.74% on SEED, 95.82 ± 1.89% on SEED-IV, and 93.35 ± 4.08% (valence) / 94.11 ± 2.98% (arousal) on DEAP. These results highlight the efficiency and flexibility of Multilayer-GTCN across varied datasets. By merging a physical proximity graph with correlation-based functional connectivity in a multilayer architecture, this study lays a foundation for scalable affective-computing systems and delivers a framework to guide upcoming advances in neural signal study.

## 1 Introduction

Emotion recognition from electroencephalography (EEG) is a rapidly advancing area in affective computing with direct relevance to mental health assessment, human–computer interaction, and neurofeedback applications (Picard et al., 2001; Cowie et al., 2001). EEG measures brain activity directly, offering an objective view of emotional state and avoiding many of the biases that burden self-report instruments (Daly et al., 2013). This objectivity is valuable for tasks such as monitoring affect during therapy and building adaptive, emotion-aware interfaces that respond to users in real time (Calvo and D’Mello, 2010). At the same time, EEG signals are intricate, non-linear, and easily corrupted by noise, which makes reliable emotion decoding technically challenging and keeps the problem squarely in active research (Alarcao and Fonseca, 2017). In the past, researchers used classic machine learning approaches such as support vector machines (Vijayakumar et al., 2020) and k-nearest neighbors, together with hand-crafted characteristics like power spectral density and wavelet coefficients (Murugappan et al., 2010). While the results were promising, these pipelines struggled to capture large-scale spatial and temporal dependencies across brain areas and seemed to get worse in noisy or fluctuating environments (Jenke et al., 2014).

Recent deep learning approaches, including CNNs and RNNs, improved feature extraction from raw EEG data (Li C. et al., 2021). However, challenges remain in modeling intricate EEG dynamics (Lin et al., 2010), driving the adoption of graph neural networks to effectively capture brain connectivity.

In recent years, the development of graph neural networks (GNNs) has made significant strides in deep learning (Monti et al., 2017a; Zhong et al., 2020), which has provided novel methodologies for addressing these challenges (Bronstein et al., 2017). GNNs are particularly well-suited for this task because they are specifically designed to process graph-structured data, which can be represented by nodes representing brain regions and edges representing their interactions (Bullmore and Sporns, 2009). GNNs excel at modeling dynamic brain connectivity crucial for emotion recognition (Song et al., 2018; Rahman et al., 2025; Wang et al., 2018).

Models integrating GNNs with techniques such as 1D CNNs and GCNs have demonstrated good performance in emotion classification, leveraging both spatial relationships and functional connectivity (Nahin et al., 2023; Kipf and Welling, 2016). SOGNN (Li J. et al., 2021) builds graphs by linking EEG channels according to their correlations. It performs reasonably, but the variability in its results suggests that key signal attributes are still being missed. STGATE (Li J. et al., 2023) improves on this with dynamic adjacency matrices and generally stronger outcomes, yet it still has difficulty capturing truly long-range interactions. ResGAT (Chao et al., 2023) focuses on functional connectivity and does well on both valence and arousal, though its use of static connectivity limits how well it adapts across conditions. GC-GCNN (Zhang et al., 2022) relies on Granger causality and also performs well, but it can overlook global dependencies and more complex spatial patterns, which curbs its effectiveness in varied settings.

In this research, we introduce the Multilayer Graph Transformer Convolutional Network (Multilayer-GTCN), an innovative architecture aimed at surpassing the constraints of current models. The architecture is distinguished by the sophisticated integration of Graph Transformer layers (Hoang et al., 2021; Vaswani et al., 2017), recognized for their proficiency in capturing global dependencies, and GCNs (Fan et al., 2024), esteemed for their capability to model local, node-specific interactions. This multilayer architecture effectively integrates both global and local patterns in EEG data (Monti et al., 2017b), offering a more advanced methodology for emotion recognition.

In addition, Multilayer-GTCN employs a dual-layered graph formulation to enhance EEG-based affect decoding by jointly modeling complementary structural and functional relations among feature nodes (Lin et al., 2023). Specifically, the framework has two layers: a physical proximity layer, which is a complete prior that makes sure all pairs are connected in the same way, and a functional connectivity layer, which is based on data-driven inter-feature correlations (Almohammadi and Wang, 2024). For each layer, adjacency matrices are constructed and converted into edge indices to enable efficient propagation while preserving the intended topology. This dual-layered mechanism captures coordinated global interactions alongside localized regularities in the feature space, allowing the model to disentangle complementary spatial–spectral cues and to mitigate variability and noise that commonly hinder robust learning. Together, the combination of structural and functional components in the Multilayer-GTCN forms a robust and adaptable emotion recognition pipeline, capable of performing well across diverse recording conditions. In summary, the major contributions of this study are as follows:

(1) We present Multilayer-GTCN, a hybrid graph model for EEG-based emotion recognition that pairs Graph Convolutional Networks (GCNs) with Graph Transformer layers and learns over two complementary graphs. By merging global context modeling through attention and stabilizing it with local structural refinement, this design delivers a practical, scalable approach to affective computing with stronger robustness, higher precision, and better adaptability.
(2) The model constructs adjacency matrices for a physical proximity layer instantiated as a complete topology providing uniform pairwise connectivity and a functional connectivity layer whose edges arise from data-driven inter-feature correlations. These matrices are converted to edge indices, enabling principled analysis of structural organization and functional interactions in the EEG feature space.
(3) The model employs Transformer attention and GCN-based neighborhood aggregation over two complementary feature space graphs, physical proximity and functional connectivity. The Transformer layers dominate by capturing global, non-local dependencies, while the GCN layer contributes supportive spatial regularization that reinforces local consistency among feature nodes. This dual-graph integration produces richer, more stable embeddings and enhances the model’s ability to decode emotional states from EEG signals.
(4) The model is validated through extensive experiments on SEED, SEED-IV, and DEAP datasets, demonstrating that global dependency modeling plays a dominant role, with local aggregation providing supportive stabilization. These results highlight the model’s effectiveness in analyzing EEG-based affective dynamics across diverse contexts.

The article is organized as follows: Section 2 details the methodology, datasets, preprocessing, feature extraction, dual-graph construction, and the Multilayer-GTCN architecture. Section 3 reports experimental results and improvements on all three datasets. Section 4 gives a summary of the main results and suggests ways to move further with the work.

## 2 Methodology

This section describes how we use EEG to recognize emotions. It includes steps like signal pre-processing, feature extraction, and building two graphs, and then it shows how we designed a Multilayer-GTCN that learns from these graphs. The model uses graph-based learning instead of physical proximity and functional connectivity to capture both global and local structure, which helps it make strong affective predictions.

### 2.1 Datasets

We evaluate the proposed GNN-based model on three widely used EEG emotion datasets: SEED, SEED-IV, and DEAP datasets. SEED (Zheng and Lu, 2015) comprises recordings from 15 participants (7 male and 8 female) collected over three sessions to capture temporal variability. Each session contains 15 trials in which participants view ~4-min film clips designed to elicit positive, neutral, or negative affect. EEG was sampled at 200 Hz from 62 channels arranged according to the international 10–20 system. SEED IV (Zheng et al., 2018) follows the same recording setup (15 participants, 62-channel EEG) but expands the label space to four emotions: neutral, sad, fear, and happy. Each of the three sessions includes 24 trials with ~2-min clips. For our study, we focus solely on the EEG modality and exclude eye movement features. DEAP (Koelstra et al., 2011) contains data from 32 participants who each watched 40 1-min music videos. The dataset provides 32-channel EEG at 128 Hz and peripheral physiological signals at 256 Hz, together with self-reports of arousal, valence, dominance, and liking on a 1–9 scale, yielding 1,280 trials in total.

### 2.2 Data preprocessing

**Figure 1:**
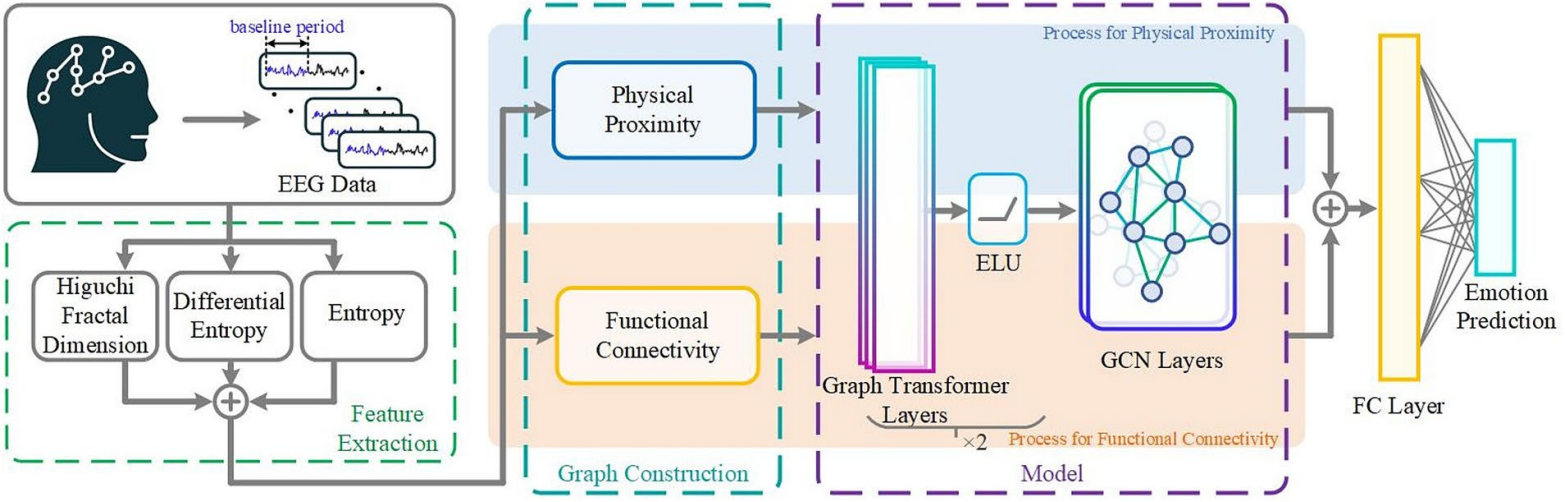
Overview of the proposed Multilayer-GTCN framework for EEG-based emotion recognition. The pipeline starts with preprocessing and feature extraction using Higuchi Fractal Dimension, Entropy measurements, and Differential entropy. Dual graphs are then generated based on physical closeness and functional connectedness. Then, they are processed using a shared Transformer-GCN architecture, which captures both global and local relationships. The outputs are combined using a fully connected (FC) layer for final emotion classification.

Preprocessing is a critical stage in EEG-based emotion recognition because it directly shapes feature quality and, in turn, model performance (Dhanaselvam and Chellam, 2023). In line with standard practice on SEED, SEED-IV, and DEAP, our pipeline addresses high dimensionality, noise, and between-session/subject variability. As shown in Figure 1, we begin with signal cleaning to ensure consistency and readiness for analysis. A 1–50 Hz band-pass filter was applied to include the lower gamma range while retaining EEG rhythms commonly associated with emotion processing, following established EEG emotion-recognition protocols (Zheng and Lu, 2015). Following filtering, z-score normalization was used to make the EEG data from different subjects and sessions comparable. Raw EEG amplitudes vary due to electrode impedance, scalp contact, or recording noise, which can mislead the model. To address this, each channel within every trial was standardized by subtracting its mean and dividing by its standard deviation. The missing samples were first replaced with the mean value of that channel within the same trial before normalization. This process equalizes scale differences while preserving the waveform’s temporal shape, allowing the model to focus on meaningful emotional fluctuations rather than raw amplitude variations. After filtering and normalization, the EEG signals from both the SEED and DEAP datasets were segmented into fixed-length analysis windows to capture emotion-related temporal patterns. For SEED, each window was 8 s long, while for DEAP, a shorter 2-s window was used to accommodate its higher sampling rate and continuous recordings. Within each window, an initial portion was designated as a baseline period, 2 s for SEED and 1 s for DEAP (Yin et al., 2021). The mean of this baseline was computed for each channel and subtracted from the entire window to obtain a baseline-corrected signal, as expressed by Equation 1,


Calibrated_segment=segment−mean(baseline) (1)

Here, *segment* represents the EEG signal for the full analysis window, and *baseline* corresponds to its initial portion. This operation does not remove the baseline samples but re-centers each window relative to its resting activity, effectively suppressing slow drifts and static offsets. The unified procedure emphasizes stimulus-induced fluctuations, improves signal-to-noise ratio, and enhances inter-subject consistency across both datasets, consistent with established affective EEG methodologies.

### 2.3 Feature extraction

Feature extraction is an essential phase subsequent to data preprocessing, designed to convert the refined and segmented EEG signals into a collection of representative features that encapsulate the fundamental patterns linked to various emotional states (Li et al., 2018). Our methodology employs three main categories of characteristics: Higuchi Fractal Dimension (HFD) (Higuchi, 1988; Shannon, 2001), entropy features (Patel and Annavarapu, 2021), and differential entropy (Zheng and Lu, 2015), to ensure that both the non-linear dynamics and spectral characteristics of EEG activity are effectively represented.

The selection of these three methods was based on their ability to capture distinct yet complementary aspects of the emotional EEG signal. HFD quantifies the non-linear temporal complexity of EEG time series, providing a compact measure of signal irregularity and self-similarity. Emotion-related brain states are often characterized by varying degrees of complexity; higher HFD values indicate richer, more irregular neural activity. For a discrete EEG time series, x(t) with t=1,2,…,N , the HFD is computed as Equation 2,


$$\mathrm{HFD}=\frac{\log(L(k))}{\log(\frac{1}{k})}$$ (2)

Where L(k) is the average curve length obtained when the signal is divided into segments separated by an interval k . Smaller k values capture fine-scale fluctuations, while larger k values reveal broader temporal patterns. Entropy-based features describe the statistical uncertainty of EEG amplitudes, quantifying the diversity and unpredictability of neural responses without assuming a specific distribution. For each channel, the signal is transformed into a normalized histogram to estimate the probability of each amplitude level. The entropy H is calculated as Equation 3,


$$\begin{array}{c} H=\sum_{i}p_{i}\log(p_{i}) \end{array}$$ (3)

Where $p_{i}$ denotes the probability of the $i^{\mathrm{th}}$ state in the EEG signal. This metric aids in quantifying the diversity and irregularity in EEG data, which signify distinct emotional states. Differential entropy (DE) characterizes band-specific power information across canonical frequency bands, delta (1–4 Hz), theta (4–8 Hz), alpha (8–13 Hz), beta (13–20 Hz), and gamma (20–30 Hz), which have well-established correlations with affective and cognitive functions. For each channel and frequency band, the EEG signal, x(t), is band-pass filtered, and the DE is computed as Equation 4,


$$\begin{array}{c} \mathrm{DE}=\log(\mathrm{var}(x_{f})+10^{-10}) \end{array}$$ (4)

Where $x_{f}$ is the filtered signal within a specific band, var(·) denotes variance, and $10^{-10}$ is a small constant added for numerical stability. This measure reflects the logarithmic energy content of the band-limited signal. The combined use of HFD , entropy, and DE ensures a holistic characterization of the EEG signal: HFD captures temporal irregularity and dynamical complexity, entropy reflects statistical diversity and neural variability, and DE encodes frequency-specific energy changes. Together, these features provide a balanced representation of both time-domain dynamics and frequency-domain power, which are crucial for robust emotion recognition. To curb redundancy and stabilize learning, we apply principal component analysis (PCA) to project features into a lower-dimensional space while retaining the key sources of variation (Abdi and Williams, 2010). This ensures efficient and effective learning in subsequent model stages, providing a robust representation of brain activity associated with diverse emotional states.

### 2.4 Graph construction

In the continuation of our methodology, we use a multilayer graph methodology, including the Physical Proximity Layer (Zhou et al., 2023) and the Functional Connectivity Layer (Fu et al., 2024), to improve emotion identification from EEG data (Cattai et al., 2022). In our implementation, both layers are constructed over feature nodes produced by the data pipeline and are shared across samples to avoid leakage between train/test splits. The physical proximity layer is realized as a complete prior that stabilizes information flow among features, whereas the functional connectivity layer encodes data-driven relations among features estimated from correlations. By combining these complementary priors, the Multilayer-GTCN captures comprehensive dependencies in the feature space, improving the accuracy and robustness of emotion identification. We then convert each adjacency matrix to edge indices by keeping its non-zero entries; combined with the node features, these indices form the graph input the model processes.

To construct the physical proximity layer, we define a binary adjacency matrix, $A_{P}\in {\mathbb{R}}^{N\times N}$ , where N is the number of feature nodes. This matrix connects every pair of distinct nodes to model dense, uniform interactions. The $\;A_{p_{\mathrm{ij}}}$ is calculated as follows Equation 5,


$$\;A_{p_{\mathrm{ij}}}=\{\begin{array}{c} 1\;\;\;\text{if}\;i\neq j\\ 0\;\;\;\text{otherwise} \end{array}$$ (5)

Where $A_{P_{\mathrm{ij}}}=1$ if feature nodes i and j are physically adjacent, and $A_{P_{\mathrm{ij}}}=0$ otherwise. This complete topology acts as a stable prior on the feature graph, promoting dense information exchange among nodes without relying on electrode geometry and providing robust support for downstream affective recognition.

On the other hand, the functional connectivity layer captures the functional relationships between signals represented in the feature space. This layer is constructed by computing Pearson correlation coefficients between feature dimensions using the available data, resulting in a weighted adjacency matrix, $A_{F}\in {\mathbb{R}}^{N\times N}$ , where N is the number of feature nodes. The matrix $\;A_{F_{\mathrm{ij}}}$ is defined as Equation 6,


$$\begin{array}{c} \;A_{F_{\mathrm{ij}}}=\frac{\mathrm{cov}(X_{i},X_{j})}{\sigma X_{i}\sigma X_{j}} \end{array}$$ (6)

**Figure 2:**
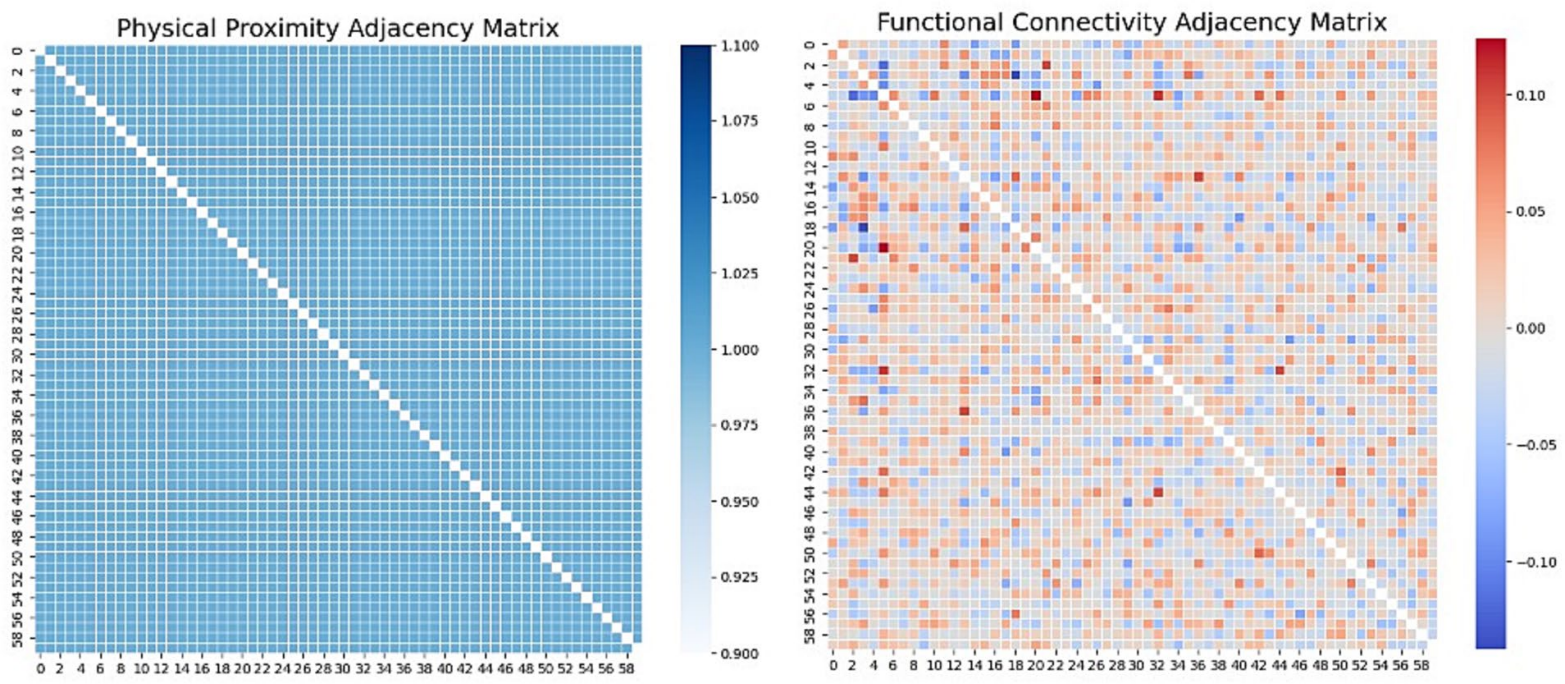
Adjacency Matrices on SEED. Left: Physical Proximity adjacency implemented as a complete graph (unit weights on all off-diagonal entries; no self-loops), indicating uniform pairwise connectivity among nodes. Right: Functional Connectivity adjacency Pearson correlation between node signals, with red denoting positive and blue denoting negative relationships (magnitude reflects correlation strength).

Where $\mathrm{cov}(X_{i},X_{j})\;$ is the covariance between the features of nodes i and j , and $\sigma X_{i}$ and $\sigma X_{j}\;$ are the standard deviations of the features of nodes i and j . To obtain a sparse binary graph suitable for GNN input, $A_{F}\;$ is the thresholder by their mean value and then converted into indices of existing edges. In Figure 2, both adjacency matrices are adjusted to ensure they have the correct dimensions, matching the number of nodes (features), and then converted into edge indices suitable for graph neural network input by identifying the non-zero elements, represented as Equation 7,


$$\begin{array}{c} \text{Edge Index}=\mathrm{np}.\text{nonzero}(A) \end{array}$$ (7)

The nodes’ features and edge indices are employed to designate the graph data for each layer, and the edge indices are reindexed to align with the train/test masks, thereby guaranteeing that the graph neural network processes the data in the appropriate format without information leakage. These graphs collectively serve as the foundation of our model’s capacity to precisely identify emotions through EEG signals.

### 2.5 Model architecture

Graph Neural Networks (GNNs) are neural architectures built to learn from graph-structured data, where information is expressed as nodes and the relationships between them as edges. This format naturally resembles settings such as social graphs, molecular structures, and brain connectivity, where dependencies are neither purely sequential nor grid-like.

Let G=(V,E) be a graph with a node set V and edge set E. Each node v∈V carries a feature vector $\;h_{v}$ . GNNs learn updated node embeddings by repeatedly mixing a node’s features with those of its neighbors. At layer k , a generic update takes the form of Equation 8,


$$\begin{array}{c} h_{v}^{\left(k\right)}=\text{AGGREGAT}E^{\left(K\right)}(h_{v}^{\left(k-1\right)},\{h_{u}^{\left(k-1\right)}:u\in N(v)\}) \end{array}$$ (8)

Where N(v) is the neighborhood of the node v , and $\text{AGGREGAT}E^{\left(K\right)}\;$ is a learnable function that aggregates information from the node v and its neighbors. $h_{v}^{\left(k-1\right)}$ represents the feature vector of node v from the previous layer, encapsulating what that node has already learned up to iteration k−1. Each neighboring node u∈N(v) contributes its own representation $h_{u}^{\left(k-1\right)},$ which encodes the information accumulated by that node in the previous layer. Through repeated aggregation across layers, the model captures both local structural dependencies (from immediate neighbors) and progressively broader, multi-hop contextual relationships within the graph.

Our model employs a hybrid graph neural network that integrates both physical proximity and functional connectivity information from EEG signals, as illustrated in Figure 1. It combines Transformer-based graph layers with GCN layers to perform the AGGREGATE operation, where the Transformer modules execute attention-weighted integration of neighbor information, and the GCN layers apply degree-normalized aggregation to stabilize and refine node embeddings. Stacking these layers across multiple stages enables the network to learn local neighborhood patterns while also capturing broader, long-range dependencies within the graph.

#### 2.5.1 Graph transformer layer

Graph Transformer layers are designed to capture long-range dependencies and global information flow in graph-structured data. In the proposed Multilayer-GTCN, these layers operate on both constructed feature space graphs, the physical proximity graph, which provides a uniform prior over all nodes, and the functional connectivity graph, which encodes correlation-based dependencies among features. Each Transformer layer uses a self-attention mechanism that helps the network focus on the most informative node–edge relationships within the graph. In practice, this means that the model learns to emphasize those feature interactions that are most relevant for recognizing emotional states. To construct the model, the node features V (Obtained from the extracted EEG features, where each node corresponds to a feature dimension rather than an electrode) and the edge indices E (Computed from the two adjacency matrices) are provided as inputs to the Transformer layers. The shared encoder applies two Transformer layers sequentially to both graphs, which promotes consistent feature learning and reduces redundancy between the physical and functional representations. In the Transformer stage, information can pass between all nodes in the graph rather than being restricted to immediate neighbors. This global message exchange helps the model recognize long-range relationships that standard convolutional filters usually cannot detect. For a given node i , the output of the Transformer layer is obtained by aggregating information from its connected neighbors according to Equation 9,


$$\begin{array}{c} h_{i}^{\prime }(\text{Transformer})=\sigma \;(\sum_{j\in N(i)}\alpha_{\mathrm{ij}}\cdot W\cdot h_{j}) \end{array}$$ (9)

Here, $\;h_{i}^{\prime }(\text{Transformer})\;$ is the updated feature vector for the node i,N(i) denotes the neighboring nodes, σ is an activation function, W is a trainable weight matrix, $h_{j}$ represents the current feature embedding, and $\alpha_{\mathrm{ij}}\;$ corresponds to the attention weight between nodes i and j . These attention coefficients are computed as Equations 10–11,


$$\begin{array}{c} \alpha_{\mathrm{ij}}=\frac{\exp(e_{\mathrm{ij}})}{\sum_{k\in N(i)}\exp(e_{\mathrm{ik}})} \end{array}$$ (10)

$$\begin{array}{c} e_{\mathrm{ij}}=\frac{{\left(Wh_{i}\right)}^{T}(Wh_{j})}{\sqrt{d_{k}}} \end{array}$$ (11)

**Figure 3:**
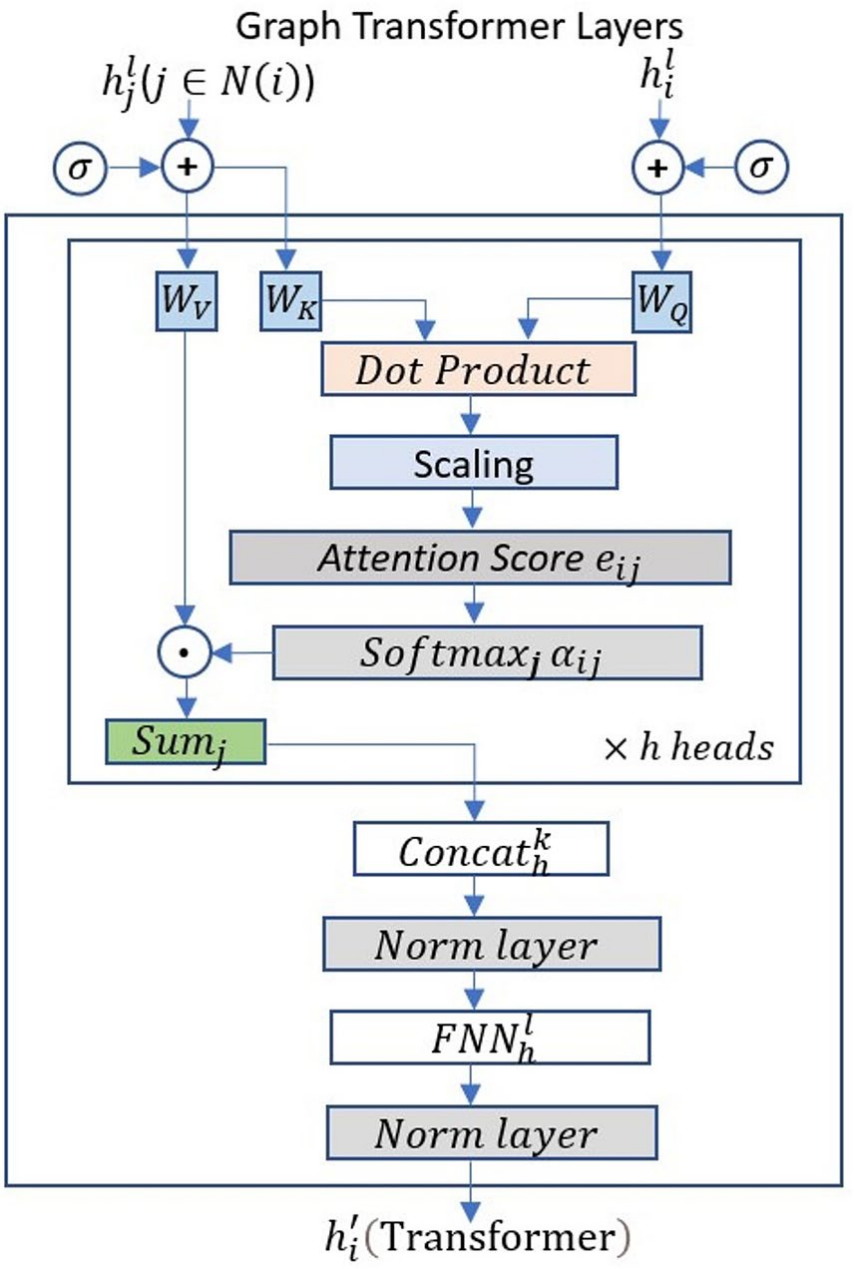
Inside the mechanism of Graph Transformer Layers. The node feature $h_{i}^{l}$ undergoes multi-head attention, where query $W_{Q}$ , key $W_{K}$ , and value $W_{V}$ matrices are learned and applied to compute attention scores $a_{\mathrm{ij}}$ between nodes. The attention-weighted neighbor features $h_{j}^{l}$ are aggregated over the neighbors j , and the multi-head outputs are concatenated and normalized to produce the updated node embedding.

Here, $d_{k}$ denotes the dimensionality of the transformed features. This scaled dot-product attention mechanism dynamically adjusts edge importance according to each trial’s cognitive state, enabling the layer to adaptively highlight dominant global patterns related to emotional processing. Each Transformer layer is followed by an ELU activation to enhance non-linear expressiveness. When combined within the shared encoder, the Graph Transformer layers provide the global reasoning foundation of the Multilayer-GTCN, upon which the subsequent GCN layer performs local aggregation. The overall structure and attention flow are illustrated in Figure 3.

#### 2.5.2 Graph convolutional network layers

While the Transformer layers are responsible for capturing global, long-range relationships across the graph, the GCN layers pay attention to the local structure for both graphs, that is, how each node exchanges information with its nearby neighbors. In our model, the GCN comes right after the Transformer in the shared encoder. This placement lets it refine and smooth the features that were first shaped by global attention, giving a more stable and locally consistent representation of the data. At each layer, a node’s features are updated by aggregating information from its neighbors with degree-based normalization, which can be written as Equation 12,


$$\begin{array}{c} h_{i}^{\prime }(\mathrm{GCN})=\sigma (\sum_{j\in N(i)}\frac{1}{c_{\mathrm{ij}}}\cdot W\cdot h_{j}) \end{array}$$ (12)

Where $h_{i}^{\prime }(\mathrm{GCN})$ is a new feature representation for node i, σ is an activation function (typically ELU ), W is a learnable weight matrix, and $c_{\mathrm{ij}}$ is a normalization constant that can be based on the degree of nodes iandj . This convolution operation aggregates signals from neighboring nodes, reinforcing spatial consistency and reducing overfitting to noise in the EEG feature space. By integrating the GCN after the Transformer layers, the model achieves a hierarchical balance. The Transformer captures dominant global dependencies, while the GCN smooths and stabilizes local patterns that reflect short-range functional connectivity and subtle emotional nuances. Together, these mechanisms complete the shared Transformer–GCN encoder, ensuring clear multi-scale representation learning across both physical and functional graphs.

#### 2.5.3 Multilayer integration

The proposed Multilayer-GTCN architecture performs integrated learning across two feature-space graphs, a physical proximity graph that provides a uniform prior over all feature nodes, and a functional connectivity graph that encodes correlation-based dependencies among features. We used the same encoder for both graphs, which includes two Transformer layers and one GCN layer. Sharing the encoder keeps the overall network smaller and makes it easier to learn features similarly from both graph assessments. The Transformer layers capture dominant global, long-range dependencies among feature nodes, modeling non-local interactions that are crucial for emotion discrimination. Each Transformer layer is followed by an ELU activation to improve non-linear expressiveness. In comparison, the GCN layer plays a smaller but still important role. It performs local aggregation that smooths and stabilizes nearby node features, helping to reinforce spatial consistency without changing the broader global patterns already established by the Transformer (Pessoa, 2017). Finally, the view-specific embeddings from the physical and functional graphs are concatenated in a late-fusion stage, and a lightweight fully connected classifier projects the fused representation into a higher-dimensional hidden space before producing the final logits. This feature space dual-graph, weight-shared Transformer–GCN design efficiently integrates both global and local dependencies, with global attention exerting a dominant influence and local aggregation providing supportive refinement, resulting in richer and more stable representations for EEG-based emotion recognition. This hierarchical integration also reflects the coexistence of distributed (global) and localized (local) affective brain mechanisms observed in cognitive and affective neuroscience (Rolls, 2018; Lindquist et al., 2012).

#### 2.5.4 Feature combination and classification

Once the Transformer and GCN layers finish processing the node features inside the shared encoder, their outputs are joined. The two graphs, the physical proximity and the functional connectivity, carry different kinds of information. In our setup, the physical graph mainly keeps the structure balanced for all nodes, while the functional graph shows how the features actually relate to one another during different trials. To integrate information from both graphs, we combine their respective outputs using Equation 13, forming a unified feature space that captures both structural topology and functional relationships.


$$\begin{array}{c} h_{\text{combined}}=[h_{i}^{\prime }(\text{physical})\;|\;h_{i}^{\prime }(\text{functional})] \end{array}$$ (13)

Where [⋅∣⋅] denotes vector concatenation along the feature dimension. After a few rounds of message passing, these merged features are sent to a fully connected layer, which gives the final prediction of the emotion class. The operation of this step can be written as Equation 14,


$$\begin{array}{c} \text{Output}=\text{Softmax}(W_{\mathrm{fc}}\cdot h_{\text{combined}}+b) \end{array}$$ (14)

Here, $W_{\mathrm{fc}}\;$ denotes the fully connected layer’s weight matrix, $h_{\text{combined}}$ is the fused representation from the Transformer and GCN branches, and b is the bias term. A final SoftMax maps the resulting logits to class probabilities over the emotion labels. This stage completes the Multilayer-GTCN construction pipeline by linking the feature-space fusion to the classifier output, thereby translating multi-scale graph representations into discrete emotional states. The design ensures the entire architecture, from dual-graph encoding to classification, is trained jointly and optimized end-to-end, fulfilling the integration strategy emphasized throughout the model.

#### 2.5.5 Loss function and optimization

The model is optimized using the negative log-likelihood (NLL) loss, which is well-suited for multi-class emotion-recognition tasks. For a single training sample i , the loss is defined as Equation 15,


$$\begin{array}{c} L=-\log p(y_{i}|x_{i}) \end{array}$$ (15)

Where $p(y_{i}|x_{i})\;$ is the predicted probability assigned to the true class. We optimize parameters with the Adam Optimizer and use a plateau-based scheduler that reduces the learning rate when validation loss stops improving. The Multilayer-GTCN is therefore trained end-to-end to jointly minimize classification error while balancing the complementary effects of Transformer-based global reasoning and GCN-based local aggregation across both physical and functional graphs. This integrated optimization process ensures that the model effectively learns multi-scale dependencies in EEG data, producing a robust and high-performing emotion-recognition system even under noisy and complex signal conditions.

To evaluate the performance of Multilayer-GTCN, we use accuracy, precision, recall, and F1-score. Accuracy is calculated as the proportion of correct predictions over the total number of samples, as defined in Equation 16,


$$\begin{array}{c} \text{Accuracy}=\frac{\text{Number of Correct Predictions}}{\text{Total Number of Samples}} \end{array}$$ (16)

Precision is calculated, according to Equation 17, as the ratio of true positives to all samples predicted as positive.


$$\begin{array}{c} \text{Precisio}n_{c}=\frac{TP_{c}}{TP_{c}+FP_{c}}\;\text{and Recal}l_{c}=\frac{TP_{c}}{TP_{c}+FN_{c}} \end{array}$$ (17)

Where $TP_{c}$ (True Positives) is the number of instances that are correctly predicted as class c ; $FP_{c}$ (False Positives) is the number of instances that are incorrectly predicted as class c ; and $FN_{c}$ (False Negatives) is the number of instances that actually belong to class c but were incorrectly predicted as a different class. F1-score, as defined in Equation 18, is calculated as the harmonic mean of precision and recall.


$$\begin{array}{c} F1_{c}=\frac{2\cdot \text{Precisio}n_{c}\cdot \text{Recal}l_{c}}{\text{Precisio}n_{c}+\text{Recal}l_{c}} \end{array}$$ (18)

These metrics provide a balanced assessment of the model’s performance, capturing both overall accuracy and class-wise performance.

## 3 Results and discussion

### 3.1 Experimental setup

The experimental configuration for the Multilayer-GTCN utilizes a subject-independent methodology in SEED, SEED IV, and DEAP datasets. The Multilayer-GTCN design has two Graph Transformer layers and one GCN layer. The first Transformer layer runs on both physical-proximity graph and functional connectivity graph, using 128 features with 8 attention heads, dropout 0.5, and ELU activation. A second Transformer block follows, keeping 8 heads and the same dropout and activation while producing 128-dimensional outputs. We apply these Transformer stages separately to the physical and the functional edge indices so that each view contributes its own pattern of interactions among nodes. Afterward, a single GCN layer aggregates neighborhood information for each graph, preserving a 128-dimensional representation and using batch normalization with ELU to stabilize and strengthen the features. The representations yielded by the Transformer and GCN stages are then passed to a fully connected module with 256 hidden units, followed by batch normalization and ELU, and finalized with a log-SoftMax layer to produce a probability distribution over emotion classes. Training uses the Adam optimizer with a learning rate of 0.0001, 5e-4 weight decay, and a CosineAnnealingLR schedule that adjusts the learning rate according to validation results. Training occurs for a maximum of 300 epochs, with early termination implemented if validation loss shows no progress for 10 consecutive epochs. A 5-fold stratified cross-validation rigorously evaluates the model’s performance, emphasizing parameters such as accuracy, F1-score, and confusion matrices. All experiments are conducted on a Windows platform using Python version 3.12.4 and the PyTorch library version 2.5.1. The trials are performed in a GPU-enabled environment to manage computational needs efficiently, ensuring the model can successfully learn from and generalize to novel, unseen participants.

### 3.2 Classification performance analysis

**Figure 4:**
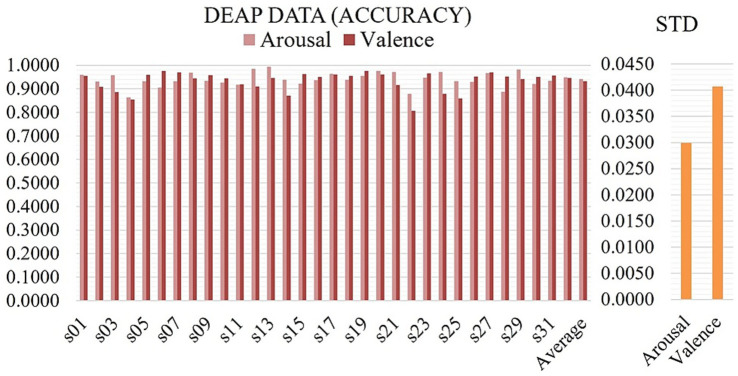
Classification performance on DEAP dataset across all 32 subjects.

In this research, we developed and tested a new graph neural network (GNN)-based model for EEG-based emotion identification using data from the SEED, SEED IV, and DEAP datasets. The objective was to accurately classify emotional states by leveraging the structural and functional properties of EEG data through graph construction and advanced neural network layers. Our model combines Transformer and Graph Convolutional layers to jointly capture global and local relationships in EEG data. This design reflects the dual organization of emotional processing in the brain, where distributed large-scale networks coordinate affective responses while localized regions, such as the prefrontal and limbic areas, contribute region-specific modulation (Pessoa, 2017; Lindquist et al., 2012; Rolls, 2018). The SEED dataset, which focuses on categorizing emotions as positive, neutral, and negative, was used as a benchmark to evaluate the model’s capacity to discriminate between these emotional states. On the SEED dataset in Table 1, our model achieved an overall accuracy of 98.24% with a standard deviation of 1.74%. The F1-score, which balances precision and recall, was similarly high at 98.24%. These findings demonstrate that the model can accurately discriminate between positive, neutral, and negative emotional states. The high F1-score implies that the model’s predictions are accurate and dependable, with minimal trade-off between false positives and false negatives. On the SEED IV dataset in Table 1, the model achieved an overall accuracy of 95.82% with an F1-score of 95.82% and a standard deviation of 1.89%. These findings demonstrate that the model can accurately discriminate between happy, sad, fear, and neutral emotional states. On the other hand, the DEAP dataset, which includes valence and arousal labels shown in Table 1 and Figure 4, enabled us to evaluate the model’s performance in a more complex emotion classification task. When tested on the DEAP dataset in Table 1, the model predicted valence with an accuracy of 93.35% and F1-score of 93.35% (standard deviation: 4.08%) and arousal with an accuracy of 94.11% and F1-score of 94.11% (standard deviation: 2.98%). All individual subject accuracies for both classes are shown in Figure 4, providing a detailed view of the model’s performance across subjects. The DEAP results are modestly lower than SEED and SEED IV, which is expected, given DEAP’s greater complexity and the finer distinctions it requires the model to make. Even so, the model tracks both valence and arousal well, capturing the nuances in these dimensions of EEG. Tables 2–3 compare Multilayer-GTCN with DGCNN, 4D-CRNN, and DBGC-ATFFNet-AFTL (Sun et al., 2022) on SEED and SEED-IV (15 subjects), reporting mean accuracy (± SD) and F1-score. On the SEED dataset, Multilayer-GTCN achieves 98.24 ± 1.74% mean accuracy with an F1 of 0.9824, outperforming DGCNN (89.39 ± 0.93%, F1 = 0.892), 4D-CRNN (94.44 ± 2.70%, F1 = 0.944), and the previously strongest DBGC-ATFFNet-AFTL (97.31 ± 1.47%, F1 = 0.972). Although the numerical gap with DBGC-ATFFNet-AFTL is approximately 1%, its impact is meaningful as Multilayer-GTCN yields consistently lower standard deviations and higher per-subject reliability, exceeding 97% accuracy for 12 of 15 subjects (2–7 and 10–15). For instance, subject 12 records an almost perfect accuracy of 99.78 ± 0.11%, and subjects 3, 4, 7, 10, and 14 also approach 99% with F1 values close to 0.99, reflecting remarkably stable model behavior. Compared with the broader variability seen in 4D-CRNN (*σ* ≈ 2.7) and DBGC-ATFFNet-AFTL (σ ≈ 1.47), Multilayer-GTCN shows a steadier performance across individuals, suggesting stronger generalization rather than tuning toward particular sessions. Beyond accuracy, this marginal gain also translates into practical robustness since, in noisy, subtle EEG emotion data, even a 1% improvement can yield many more correctly recognized emotional states. By combining Transformer attention for global dependencies with GCN propagation for structural topology, Multilayer-GTCN achieves smoother performance and lower variance across subjects. In the SEED-IV dataset, where inter-session differences are more pronounced, the strength of the proposed model becomes clearer. Multilayer-GTCN attains 95.82 ± 1.89% accuracy with an F1-score of 0.9582, outperforming DBGC-ATFFNet-AFTL (89.97 ± 2.85%, F1 = 0.898), 4D-CRNN (87.75 ± 2.72%, F1 = 0.876), and DGCNN (83.81 ± 9.41%, F1 = 0.830). Even though the gain in accuracy is relatively small, it still represents a meaningful improvement. The consistency of results across participants indicates that the model adapts well to subject variability and maintains reliable learning dynamics, a sign of both robust generalization and a well-balanced architecture.

**Table 1:** Performance of the proposed model on SEED, SEED IV, and DEAP datasets.

Data sets		F1 Score (%)	Accuracy (%)	Std (%)
SEED		98.24	98.24	1.74
SEED IV		95.82	95.82	1.89
DEAP	Arousal	94.11	94.11	2.98
	Valence	93.35	93.35	4.08

**Table 2:** The overall comparison of classification performance on SEED dataset across 15 subjects.

Subject	DGCNN (Song et al., 2018)		4D-CRNN (Shen et al., 2020)		DBGC-ATFFNet- AFTL (Sun et al., 2022)		Multilayer-GTCN(Ours)	
	Acc ± Std (%)	F1	Acc ± Std (%)	F1	Acc ± Std (%)	F1	Acc ± Std (%)	F1
1	89.39 ± 0.93	0.892	93.75 ± 0.93	0.936	97.41 ± 2.46	0.973	94.40 ± 2.43	0.9444
2	80.00 ± 1.95	0.799	87.97 ± 0.71	0.879	94.81 ± 0.79	0.947	98.43 ± 0.90	0.9844
3	83.85 ± 1.34	0.838	91.69 ± 1.11	0.916	95.93 ± 0.65	0.959	99.10 ± 0.34	0.9910
4	94.01 ± 1.87	0.939	97.05 ± 1.15	0.970	98.05 ± 1.01	0.980	99.05 ± 0.25	0.9906
5	85.12 ± 1.09	0.850	92.33 ± 1.23	0.923	97.49 ± 1.02	0.974	98.60 ± 0.18	0.9860
6	91.45 ± 0.78	0.913	94.84 ± 1.28	0.948	98.08 ± 0.66	0.980	98.82 ± 0.27	0.9883
7	91.45 ± 1.41	0.913	94.66 ± 0.66	0.946	97.96 ± 0.72	0.979	99.44 ± 0.21	0.9944
8	87.77 ± 1.74	0.876	92.54 ± 1.08	0.924	95.82 ± 0.31	0.957	95.97 ± 0.83	0.9597
9	94.37 ± 1.66	0.943	97.14 ± 0.81	0.971	97.19 ± 0.56	0.961	96.08 ± 0.55	0.9608
10	82.99 ± 1.50	0.827	93.75 ± 1.29	0.936	95.43 ± 5.60	0.953	99.10 ± 0.25	0.9910
11	92.10 ± 0.67	0.920	94.52 ± 1.39	0.945	97.58 ± 0.55	0.976	98.66 ± 0.17	0.9865
12	90.04 ± 1.99	0.900	95.63 ± 0.71	0.956	98.47 ± 0.31	0.984	99.78 ± 0.11	0.9978
13	90.66 ± 1.04	0.905	95.25 ± 1.69	0.952	97.52 ± 0.49	0.975	98.38 ± 0.30	0.9836
14	92.60 ± 1.25	0.925	96.05 ± 0.72	0.960	98.70 ± 0.35	0.987	99.16 ± 0.32	0.9917
15	97.79 ± 0.46	0.977	99.44 ± 0.48	0.994	99.23 ± 0.36	0.992	98.60 ± 0.36	0.9861
Ave	89.39 ± 0.93	0.892	94.44 ± 2.70	0.944	97.31 ± 1.47	0.972	98.24 ± 1.74	0.9824

**Table 3:** The overall comparison of classification performance on SEED IV dataset across 15 subjects.

Subject	DGCNN (Song et al., 2018)		4D-CRNN (Shen et al., 2020)		DBGC-ATFFNet- AFTL (Sun et al., 2022)		Multilayer-GTCN(Ours)	
	Acc ± Std (%)	F1	Acc ± Std (%)	F1	Acc ± Std (%)	F1	Acc ± Std (%)	F1
1	69.44 ± 5.37	0.684	83.24 ± 4.05	0.841	87.84 ± 3.43	0.878	84.88 ± 3.58	0.8565
2	91.55 ± 9.17	0.914	90.42 ± 1.20	0.903	90.66 ± 2.17	0.907	96.14 ± 1.92	0.9614
3	92.71 ± 1.75	0.925	87.60 ± 2.36	0.876	89.07 ± 1.11	0.896	97.38 ± 1.59	0.9738
4	94.24 ± 2.85	0.943	89.95 ± 4.25	0.899	91.54 ± 3.02	0.914	97.07 ± 1.68	0.9708
5	79.31 ± 8.26	0.791	85.48 ± 2.82	0.85	87.48 ± 1.47	0.871	96.60 ± 1.81	0.9660
6	85.44 ± 8.28	0.854	88.19 ± 2.99	0.881	90.43 ± 2.77	0.903	96.61 ± 1.80	0.9661
7	78.48 ± 16.19	0.768	86.42 ± 2.62	0.861	90.02 ± 4.03	0.900	96.60 ± 1.81	0.9660
8	88.83 ± 3.78	0.886	87.95 ± 3.82	0.878	91.42 ± 1.35	0.911	96.45 ± 1.85	0.9645
9	92.22 ± 2.69	0.922	80.78 ± 2.23	0.906	91.77 ± 4.13	0.915	97.07 ± 1.68	0.9707
10	80.25 ± 11.25	0.801	87.71 ± 2.06	0.875	92.48 ± 1.65	0.923	98.30 ± 1.29	0.9830
11	65.45 ± 11.60	0.602	85.25 ± 3.47	0.85	85.84 ± 1.65	0.854	91.36 ± 2.80	0.9137
12	73.22 ± 17.06	0.703	83.72 ± 1.11	0.835	84.14 ± 2.91	0.838	98.30 ± 1.29	0.9830
13	86.48 ± 6.24	0.863	88.77 ± 2.34	0.887	90.83 ± 1.94	0.905	96.76 ± 1.77	0.9676
14	82.83 ± 11.98	0.828	86.26 ± 2.62	0.862	89.54 ± 2.68	0.891	95.68 ± 2.03	0.9568
15	96.70 ± 1.59	0.966	93.71 ± 2.06	0.935	96.59 ± 1.82	0.961	98.15 ± 1.34	0.9802
Aver	83.81 ± 9.41	0.83	87.75 ± 2.72	0.876	89.97 ± 2.85	0.898	95.82 ± 1.89	0.9582

### 3.3 Comparison with existing model

Table 4 provides a comprehensive comparison of several graph neural network models used on the SEED dataset, emphasizing the graph building methodologies used and their associated accuracies. The SEED dataset, often used for emotion recognition using EEG signals, is greatly influenced by how these models generate graphs from the data, thereby impacting their effectiveness. SOGNN (Li J. et al., 2021), using EEG channel correlations for graph edges, achieves 86.81% accuracy with a 5.79% standard deviation, indicating that plain functional correlation misses key cues for reliable emotion decoding. RGNN (Zhong et al., 2020), which also relies on functional connectivity, reports 85.30% accuracy with a 6.72% standard deviation, evidence that its performance varies with the data. STGATE (Li J. et al., 2023) introduces a dynamic adjacency that lets the graph structure adapt to the signal, improving accuracy to 90.37% and underscoring the value of modeling time-varying structure in EEG. MDGCN-SRCNN (Bao et al., 2022) combines functional connectivity with GCN and spatial recurrent convolutional neural network (SRCNN), achieving 95.08% accuracy by effectively capturing spatial and temporal features. This model proficiently encapsulates both spatial and temporal characteristics, resulting in enhanced performance. FGCN (Li M. et al., 2023) amalgamates topological, functional, and causal graphs, attaining an accuracy of 94.1%, underscoring the benefit of encapsulating several dimensions of EEG data. Our suggested approach, the Multilayer-GTCN approach, integrates physical proximity and functional connectivity for graph construction, combining structural regularization and signal interdependencies. Achieving 98.24 ± 1.74% accuracy, it is exceptional compared to other models, demonstrating that incorporating multiple views enhances data representation and significantly improves emotion recognition accuracy.

**Table 4:** Accuracy comparative analysis of multilayer-GTCN models on SEED dataset.

SEED data		
Model	Graph construction	Accuracy ± std. (%)
SOGNN (Li et al., 2021b)	Functional correlation	86.81 ± 5.79
RGNN (Zhong et al., 2020)	Functional connectivity	85.30 ± 6.72
STGATE (Li et al., 2023a)	Dynamic adjacency matrix	90.37/−
MDGCN-SRCNN (Bao et al., 2022)	Functional connectivity	95.08/−
DGCNN (Song et al., 2018)	Functional connectivity	89.39/−
FGCN (Li et al., 2023b)	Topological graph construction, Functional graph construction, Causal graph construction	94.10/−
CGCNN (Kong et al., 2022)	Causal construction	93.36/−
GCNN (Lin et al., 2023)	Functional connectivity	90.22/−
Multilayer- GTCN (Ours)	Physical proximity and functional connectivity	98.24 ± 1.74

Table 5 compares GNN models on the DEAP dataset, focusing on their graph construction methods and accuracies for valence and arousal. ResGAT (Chao et al., 2023) employs functional connectivity to find links between EEG data. It has 89.26% accuracy for valence and 87.06% accuracy for arousal. GC-GCNN (Zhang et al., 2022) estimates directed influences with Granger causality, reporting 89.48% for valence and 90.11% for arousal. Conversely, P-GCNN (Wang et al., 2019), a functional connectivity approach, exhibits a latency of 73.31% (valence) and 77.03% (arousal), suggesting that it is incapable of accurately capturing the complexity of the signal. GCNN (Lin et al., 2023), which is also based on functional connectivity, exhibits superior performance, achieving arousal at 91.00% and valence at 90.74%. An advanced methodology that incorporates spatiotemporal and frequency-space characteristics is presented by Mul-AT-RGCN (Chen et al., 2022), which achieves enhanced accuracies of 93.19% for valence and 91.82% for arousal. The Multilayer-GTCN model, which combines physical proximity with functional connectivity, surpasses all other models with accuracies of 93.35 ± 4.08% for valence and 94.11 ± 2.98% for arousal. This exceptional result highlights the efficacy of integrating structural and functional information in graph building, providing a more thorough representation of EEG data that significantly improves emotion identification accuracy.

**Table 5:** Accuracy comparative analysis of multilayer-GTCN models on DEAP dataset.

DEAP data			
Model	Graph construction	Valence (Acc ± std) %	Arousal (Acc ± std) %
ResGAT (Chao et al., 2023)	Functional Connectivity	89.26/−	87.06/−
GC-GCNN (Zhang et al., 2022)	Granger causality	89.48/−	90.11/−
P-GCNN (Wang et al., 2019)	Functional connectivity	73.31/−	77.03/−
GCNN (Lin et al., 2023)	Functional connectivity	90.74/−	91.00/−
Mul-AT-RGCN (Chen et al., 2022)	Spatiotemporal features and frequency–space features	93.19/−	91.82/−
Multilayer-GTCN (Ours)	Physical proximity and functional connectivity	93.35 ± 4.08	94.11 ± 2.98

### 3.4 Ablation study

**Figure 5:**
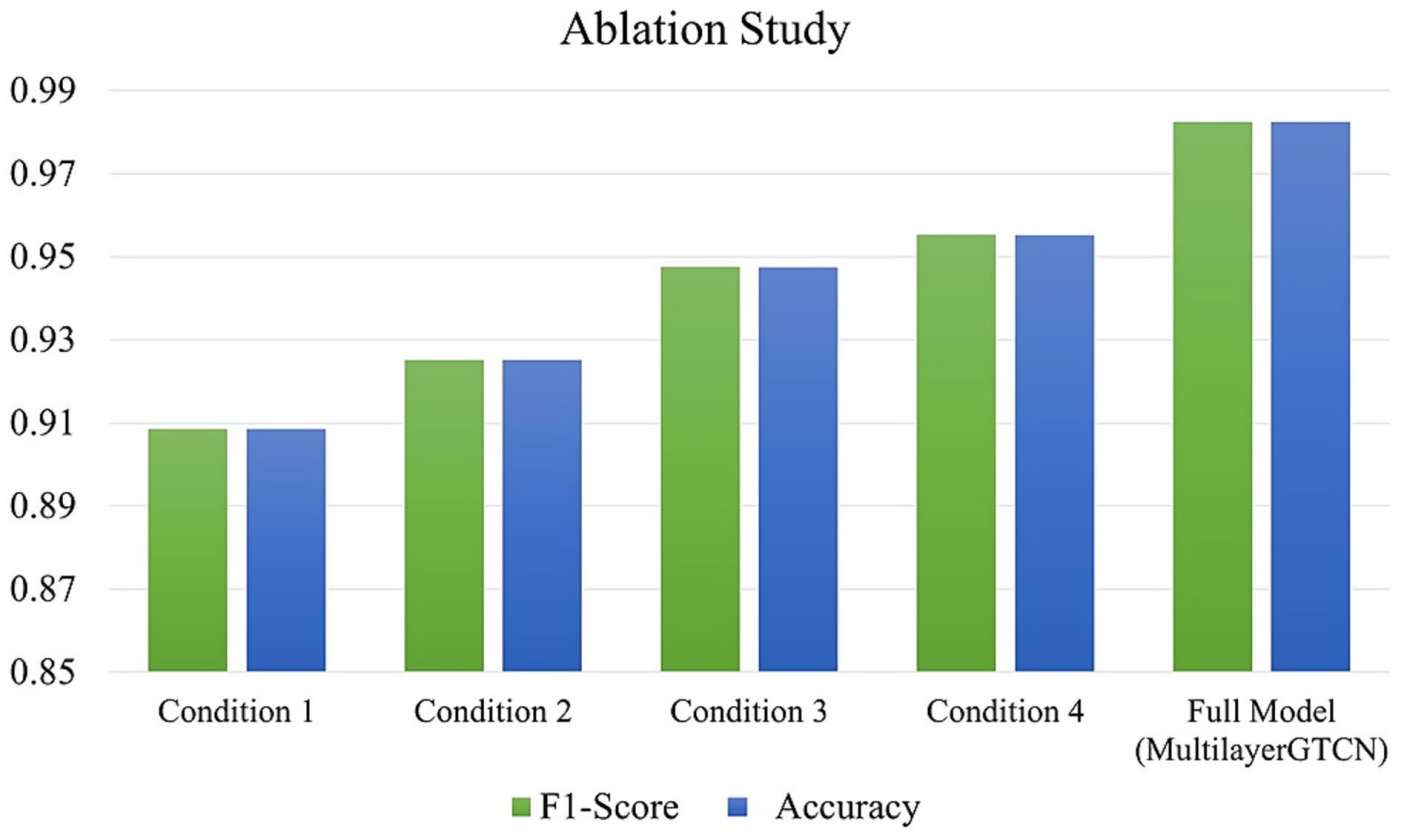
Impact of Ablation study on the SEED dataset showing F1-score and Accuracy for different model configurations.

**Figure 6:**
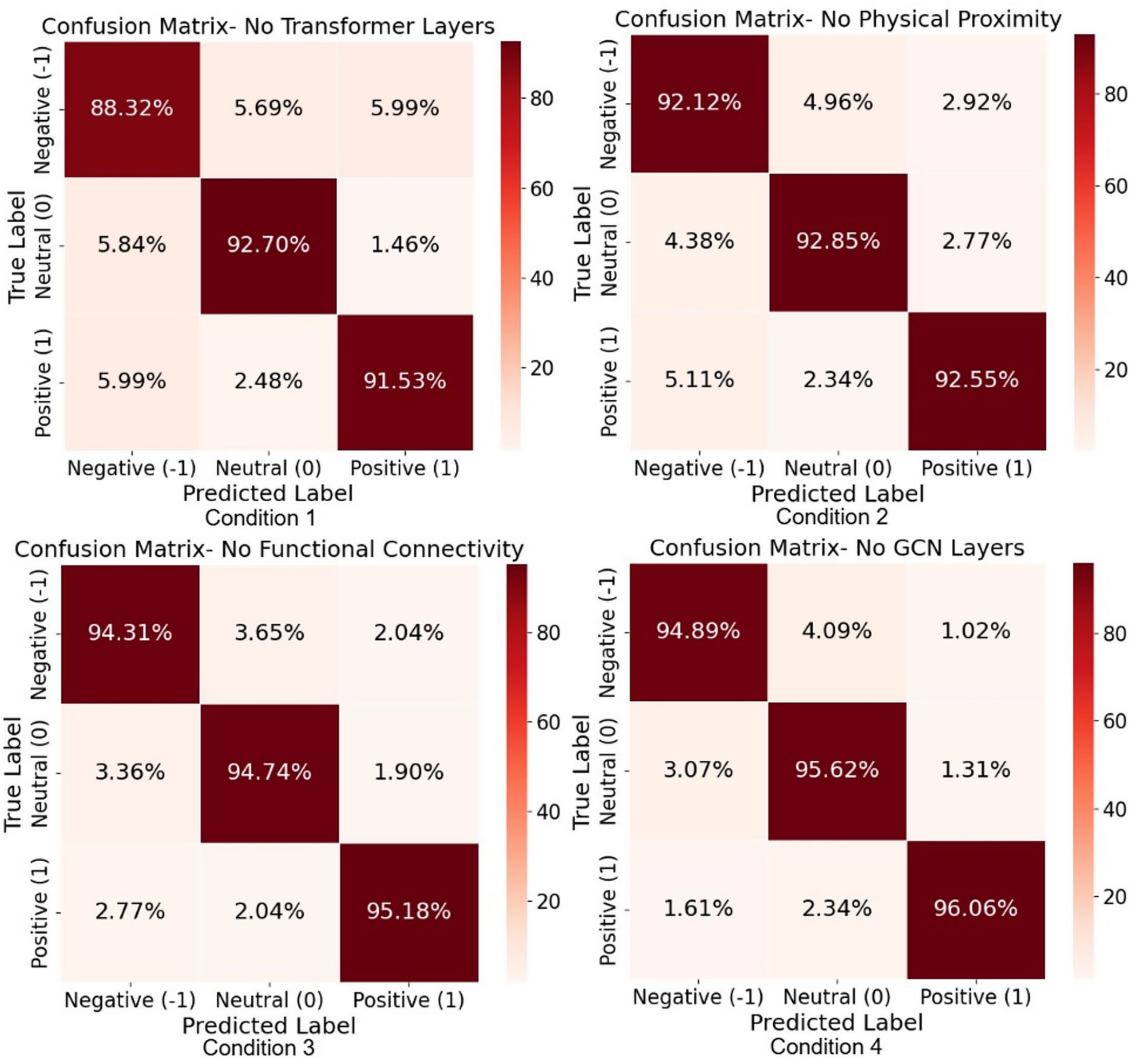
Confusion matrices from the ablation study on the SEED dataset showing classification performance for models without (i) Transformer layers, (ii) physical proximity, (iii) functional connectivity, and (iv) GCN layers. Performance degrades most notably when the Transformer layer is removed.

To evaluate the contribution of each component in our proposed Multilayer-GTCN architecture, we conducted a comprehensive ablation study on the SEED dataset under five distinct experimental configurations. The results of summary metrics are reported in Figure 5 (F1-score and Accuracy) and confusion matrices in Figure 6. The five evaluated conditions are as follows: (1) Exclusion of the Transformer layer, designed to investigate the effect of removing the global self-attention mechanism responsible for modeling long-range temporal dependencies in EEG sequences. This configuration resulted in the lowest performance among all variants, with an F1-score of 90.86% and an Accuracy of 90.85%, confirming the dominant role of the Transformer layers in capturing global dependencies. (2) Exclusion of the physical proximity graph, aimed at examining the significance of structural regularization among feature nodes. Performance declined to an F1-score of 92.52% and an Accuracy of 92.51%, indicating the utility of spatial topology in enhancing feature representation. (3) Without functional connectivity, removing the correlation-based edges (Pearson) dropped performance to 94.74% for both F1 and Accuracy, underscoring the value of functional relations in the graph. (4) Without the GCN layer, excluding localized neighborhood aggregation, resulted in a moderate decline, with 95.52% of F1-score and Accuracy, suggesting that local dependencies contribute to spatial coherence but have a less pronounced influence compared to global modeling. (5) Full Multilayer-GTCN, using all components such as Transformer, GCN, physical proximity, and functional connectivity, yielded the best results, 98.24% for both F1 and Accuracy. Overall, each component contributes meaningfully to the model’s performance, though the Transformer layers have a more pronounced influence. While the GCN layers provide a moderate contribution by refining local dependencies and stabilizing the model, the majority of the performance gain comes from the Transformer’s ability to capture global, non-local relationships. This finding suggests that, for emotion recognition in EEG data, global dependencies play a dominant role, with local dependencies providing supportive, stabilizing effects.

### 3.5 The confusion matrix analysis

**Figure 7:**
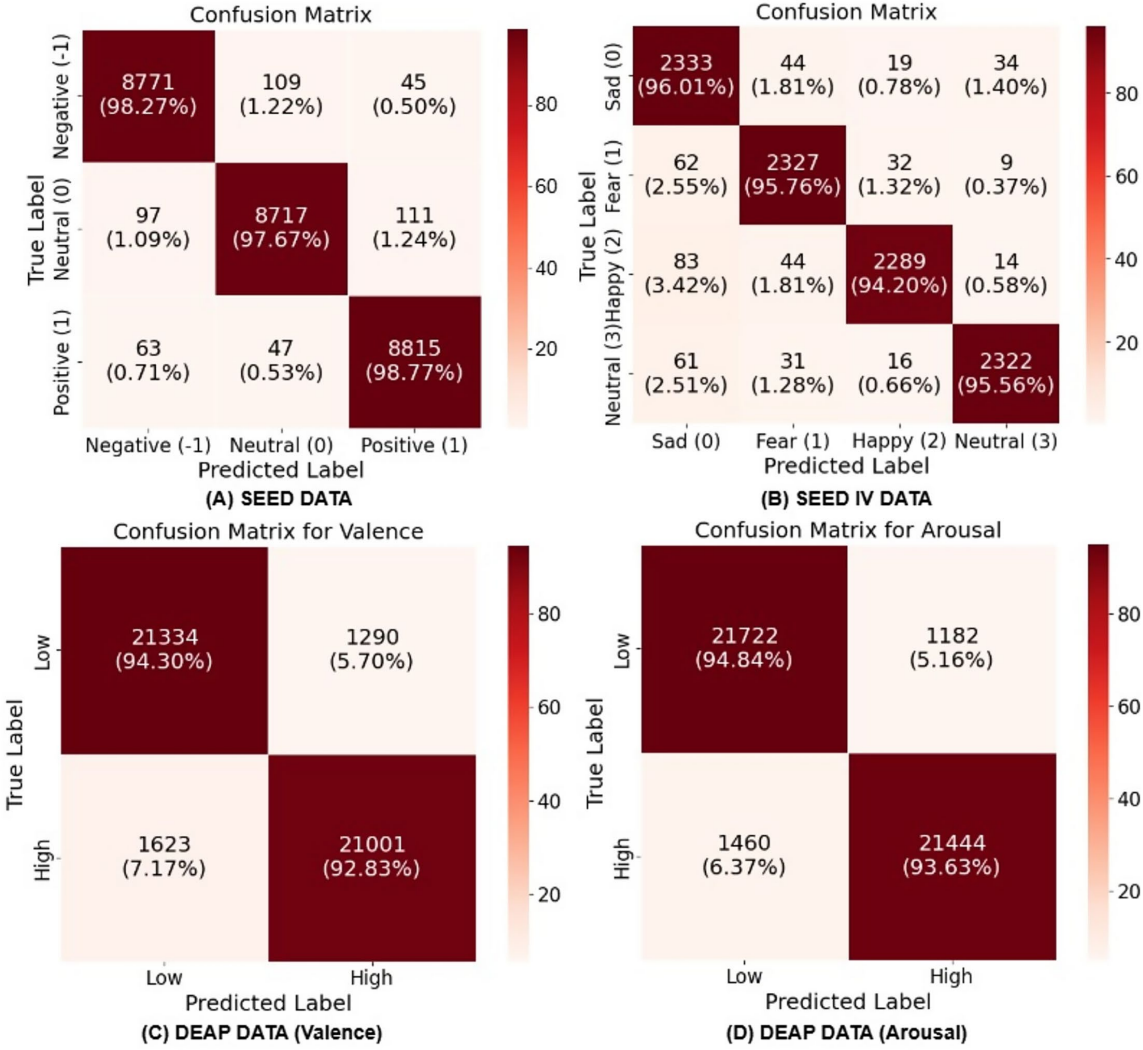
Confusion matrices showing Multilayer-GTCN’s classification performance: **(A)** SEED (three-class), **(B)** SEED IV (four-class), **(C)** DEAP Arousal (binary), **(D)** DEAP Valence (binary). Each matrix presents raw counts and normalized percentages, with diagonal elements indicating correct classifications and off-diagonal elements showing misclassifications.

Figure 7 shows confusion matrices for Multilayer-GTCN on SEED, SEED IV, and DEAP across four subfigures. The SEED matrix in Figure 7A exhibits strong diagonal dominance across all emotional classes, reflecting precise and consistent classification of negative, neutral, and positive states. Off-diagonal cells are rare, not more than 1.22%, which points to minimal pairwise confusion and clear separation among the three classes. This is consistent with the overall accuracy of 98.24% (SD 1.74%) reported in Table 1 and demonstrates that the Multilayer-GTCN model effectively distinguishes emotional classes with high reliability. In SEED-IV, Figure 7B, the diagonal remains uniformly high across sad, fear, happy, and neutral, with most per-class accuracies exceeding 94%. Errors are dispersed and small, suggesting that the model generalizes well from ternary to finer-grained, four-class labeling without concentrating mistakes in any single confusion pair. For DEAP arousal, Figure 7C, correct predictions dominate the diagonal with Low and High classes, and limited cross-class confusion with Low predicted as High at 5.16% and High predicted as Low at 6.37%. Valence, Figure 7D, shows a similar structure with Low and High classes, with slightly higher asymmetry in misclassifications (Low predicted as High at 5.70% and High predicted as Low at 7.17%), consistent with the generally subtler boundaries often observed for valence compared to arousal. Across datasets and label granularities, the confusion matrices show strong diagonal dominance with minimal off-diagonal mass, indicating consistent class-wise performance and limited error propagation. This evidence corroborates that the dual-graph design, comprising a complete physical-proximity prior together with correlation-based functional connectivity, supports complementary global (Transformer) and local (GCN) discrimination and yields robust predictions on SEED, SEED-IV, and DEAP. Although residual confusions are most visible in the valence dimension, they remain modest relative to the diagonal, underscoring Multilayer-GTCN’s stability and generalization across heterogeneous settings.

### 3.6 T-SNE visualization

**Figure 8:**
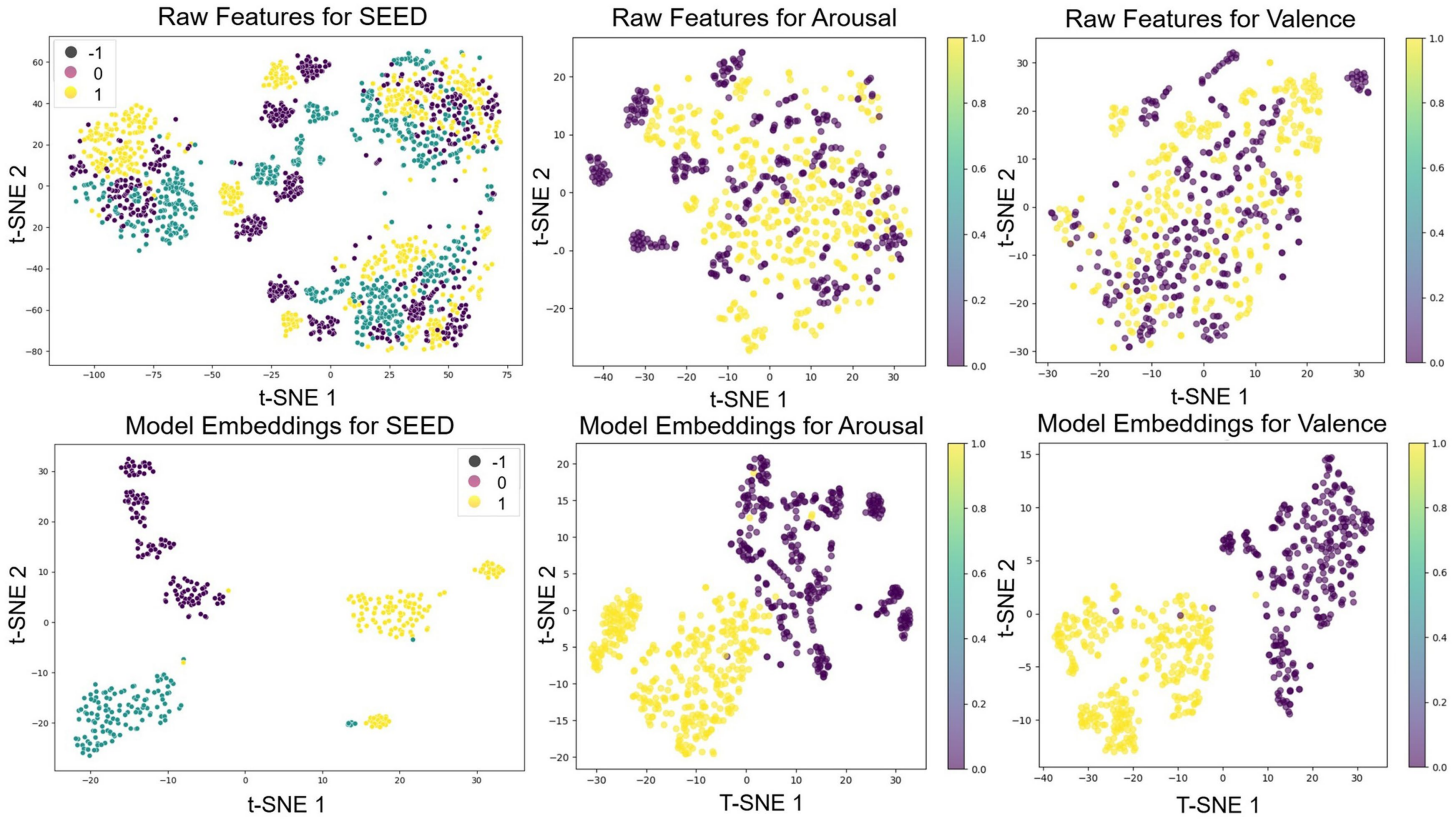
t-SNE visualization comparing raw EEG data and Multilayer-GTCN embeddings for emotion classification.

Figure 8 presents three t-SNE subfigures that evaluate how Multilayer-GTCN improves feature separability for EEG emotion classification on the SEED and DEAP datasets. In this visualization, t-SNE is applied to the latent feature vectors (256 features) obtained before the final classifier layer of Multilayer-GTCN. Each vector is formed by concatenating the 128-feature physical-graph embedding and the 128-feature functional-graph embedding. For each dataset (SEED or DEAP), the embeddings from the corresponding test set are stacked into an N × 256 matrix and then reduced to two dimensions using t-SNE for visualization. In Figure 8A SEED (positive, neutral, and negative), the top-left plot of raw EEG features shows extensive overlap among the three classes, indicating that the unprocessed representation offers little class separation. In contrast, the bottom-left plot of Multilayer-GTCN embeddings forms three compact clusters with minimal overlap, showing that the model extracts features that clearly separate the emotion label. In Figure 8B DEAP arousal (high vs. low), the top-middle plot of raw features displays substantial mixing between the two arousal levels. The bottom-middle plot shows Multilayer-GTCN embeddings with a visibly cleaner split, reflecting that the network captures patterns aligned with arousal. In Figure 8C DEAP valence (high vs. low), the upper right plot of raw characteristics demonstrates significant overlap between classes. The bottom-right plot, which shows the learned embeddings, separates valence into two clear groups, reflecting better class discrimination. Collectively, the three plots show that Multilayer-GTCN transforms overlapping raw features into compact but well-separated clusters. The improved separability points to stronger learned representations and sharper decision boundaries, which in turn support better performance on SEED’s discrete labels and on DEAP’s arousal and valence tasks.

### 3.7 Performance analysis

**Figure 9:**
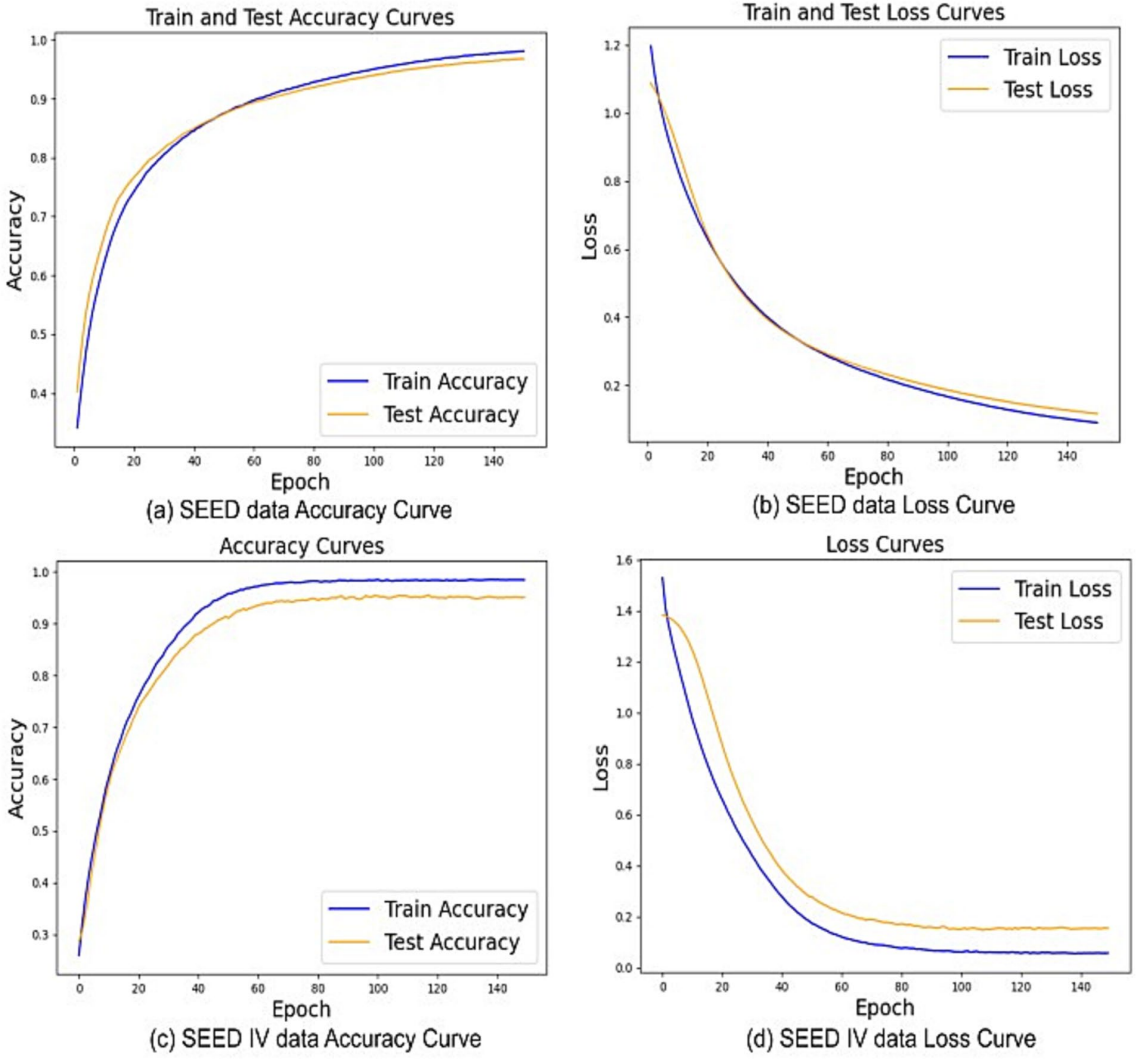
The Multilayer-GTCN model’s performance on SEED and SEED-IV datasets. **(A,B)** present accuracy and loss curves for SEED, while **(C,D)** show accuracy and loss curves for SEED-IV.

Figure 9 traces the learning behavior of Multilayer-GTCN on the SEED and SEED-IV datasets and shows how accuracy and loss evolve over training. On SEED, the accuracy curve in Figure 9A climbs rapidly during the first few epochs for both training and test splits, then rises more gradually and settles near 98%. The two curves remain tightly aligned across the entire schedule, which is typical of a model, that is, learning features that transfer to unseen data rather than memorizing the training set. Figure 9B provides the complementary view through the loss curves. Training and test loss fall steeply at the outset, then flatten as optimization converges, and their trajectories are nearly parallel, a sign of stable updates and consistent behavior across folds. The SEED-IV results follow the same pattern. In Figure 9C, accuracy jumps early, surpasses 97% at roughly 60 epochs, and then plateaus, with the test curve closely tracking the training curve. Figure 9D shows smooth, monotonic declines in loss for both splits that taper toward the end of training, mirroring the accuracy trends. Taken together, these four plots indicate steady convergence, a small and persistent gap between training and test, and no visible overfitting. In practical terms, Multilayer-GTCN learns quickly, reaches high accuracy, and maintains low, decreasing loss on both datasets. The close alignment of training and test trajectories supports the conclusion that the model generalizes reliably from EEG training data to held-out folds and that the optimization setup and regularization are well matched to the task.

## 4 Discussion

Multilayer-GTCN approaches EEG-based emotion recognition by combining two complementary views of the data within a dual-graph formulation. A complete physical-proximity graph provides a stable prior over the feature nodes, while a functional connectivity graph derived from correlations supplies data-adaptive relationships. Graph Transformer layers operate on each graph to model long-range interactions, and a GCN layer emphasizes neighborhood structure. Together, these components yield representations that capture both global interactions and local regularities, improving affect decoding without relying on a single inductive bias. The empirical results support this design across datasets and label granularities. On SEED, the model attains 98.24% ± 1.74% accuracy over 15 subjects (Table 1). The confusion matrix in Figure 7A shows clear class separation; t-SNE in Figure 8A reveals compact clusters for all three classes and learning curves in Figures 9A,B indicate stable convergence with a small train–test gap. The ablation study (Figures 5–6) confirms that both graph views and layer types contribute meaningfully, though the performance gain is primarily driven by the Graph Transformer, with the GCN providing moderate but complementary improvements that enhance local consistency and model stability. These findings are consistent with cognitive neuroscience evidence that emotion arises from both distributed and localized neural processes. Large-scale networks such as prefrontal–temporal and inter-hemispheric pathways integrate affective information globally, while regions such as the amygdala and anterior cingulate cortex support local emotional responses (Pessoa, 2017; Lindquist et al., 2012; Rolls, 2018). Although the nodes in both the physical and functional graphs represent feature dimensions rather than individual electrodes, the extracted features originate from EEG activity recorded across standardized scalp regions. Prior work highlights frontal and temporal areas reflected in channels such as F3/F4, F7/F8, and T7/T8 as central to affective processing. In our model, the physical graph enables global interaction among all feature-derived regions, while the functional graph captures stronger correlations among features originating from these emotion-related areas, consistent with established neuroscience evidence. The stronger effect of the Transformer reflects the role of global coordination, whereas the GCN’s contribution aligns with localized coherence, jointly supporting multiscale modeling of EEG-based emotion dynamics. On SEED-IV, Multilayer-GTCN reaches 95.82% ± 1.89% accuracy with 95.82% F1-score across all subjects (Table 3). The confusion matrix in Figure 7B remains diagonally dominant for sad, fear, happy, and neutral, and the accuracy/loss profiles in Figures 9C,D again show smooth optimization and strong generalization. On DEAP, performance remains high for dimensional labels, with 94.11% ± 2.98% accuracy for arousal and 93.35% ± 4.08% for valence (Table 1), and corresponding F1-score of 94.11 and 93.35%. Confusion matrices (Figures 7C,D) and t-SNE plots (Figures 8B,C) indicate well-separated classes after embedding, despite the dataset’s complexity. Comparisons in Table 4 (SEED) and Table 5 (DEAP) show that Multilayer-GTCN matches or surpasses established baselines. Integrating a physical prior with functional connectivity and coupling Transformer-based global reasoning (dominant) with GCN-based local aggregation (complementary) provides a reliable, generalizable pathway for modeling multidimensional EEG dynamics in emotion recognition, with direct relevance to neuroscience, mental health applications, and human–computer interaction.

## 5 Conclusion

In this study, we propose the Multilayer-GTCN, an innovative method for EEG-based emotion recognition that effectively integrates physical proximity and functional connectivity through a dual-graph framework. The model effectively couples Transformer layers, which capture dominant long-range dependencies, with Graph Convolutional Networks (GCNs) that provide complementary local aggregation to enhance spatial coherence within the EEG data. The dual-graph design of our model enables it to capture the complex connections among EEG signals, resulting in better emotion classification accuracy. After testing on datasets such as SEED, SEED-IV, and DEAP, our model consistently performed well, effectively distinguishing emotions across different categories, and the performance was reliable, showing great potential for emotion recognition tasks. These results suggest that emotion-related EEG dynamics are largely governed by global coordination patterns, with local structure contributing to stability and physiological interpretability. This ability to handle both the structural and functional aspects of EEG data makes the model reliable for real-world emotion recognition applications. Moving forward, we plan to refine the architecture by exploring advanced graph neural network techniques, adding more physiological signals to boost precision, and working on real-time emotion recognition capabilities. This research is a significant step advancing in the field of affective computing, with promising applications in mental health monitoring, human–computer interaction, and emotion recognition systems.

## Data Availability

The datasets used in this study (SEED, SEED-IV, and DEAP) are publicly available and have been appropriately cited in the Datasets section of this article. The SEED dataset can be accessed at http://bcmi.sjtu.edu.cn/~seed/, the SEED-IV dataset can be accessed at: http://bcmi.sjtu.edu.cn/~seed/, and the DEAP dataset is available at: https://www.kaggle.com/datasets/manh123df/deap-dataset.
